# Clinical management following self-harm in a UK-wide primary care cohort

**DOI:** 10.1016/j.jad.2016.03.013

**Published:** 2016-06

**Authors:** Matthew J. Carr, Darren M. Ashcroft, Evangelos Kontopantelis, David While, Yvonne Awenat, Jayne Cooper, Carolyn Chew-Graham, Nav Kapur, Roger T. Webb

**Affiliations:** a Centre for Mental Health and Safety, Institute of Brain, Behaviour and Mental Health, University of Manchester, UK; b Centre for Pharmacoepidemiology and Drug Safety, Manchester Pharmacy School, University of Manchester, UK; c NIHR Greater Manchester Primary Care Patient Safety Translational Research Centre, UK; d Centre for Health Informatics, Institute of Population Health, University of Manchester, UK; e NIHR School for Primary Care Research, University of Manchester, UK; f School of Psychological Sciences, University of Manchester, UK; g Research Institute of Primary Care and Health Sciences, Keele University, UK; h Manchester Mental Health and Social Care Trust, UK

**Keywords:** CI, Confidence Interval, CPRD, Clinical Practice Research Datalink, FHSA, Family Health Services Authority, GP, General Practitioner, IMD, Index of Multiple Deprivation, LSOA, Lower-Layer Super Output Area, MHRA, Medicines and Healthcare products Regulatory Agency, NHS, National Health Service, NICE, National Institute for Health and Clinical Excellence, SSRI, Selective Serotonin Reuptake Inhibitor, UK, United Kingdom, YLL, Years of Life Lost, Self-harm, Primary care, Diagnoses, Referrals, Medication

## Abstract

**Background:**

Little is known about the clinical management of patients in primary care following self-harm.

**Methods:**

A descriptive cohort study using data from 684 UK general practices that contributed to the Clinical Practice Research Datalink (CPRD) during 2001–2013. We identified 49,970 patients with a self-harm episode, 41,500 of whom had one complete year of follow-up.

**Results:**

Among those with complete follow-up, 26,065 (62.8%, 62.3–63.3) were prescribed psychotropic medication and 6318 (15.2%, 14.9-15.6) were referred to mental health services; 4105 (9.9%, CI 9.6–10.2) were medicated without an antecedent psychiatric diagnosis or referral, and 4,506 (10.9%, CI 10.6–11.2) had a diagnosis but were not subsequently medicated or referred. Patients registered at practices in the most deprived localities were 27.1% (CI 21.5–32.2) less likely to be referred than those in the least deprived. Despite a specifically flagged NICE '*Do not do*’ recommendation in 2011 against prescribing tricyclic antidepressants following self-harm because of their potentially lethal toxicity in overdose, 8.8% (CI 7.8-9.8) of individuals were issued a prescription in the subsequent year. The percentage prescribed Citalopram, an SSRI antidepressant with higher toxicity in overdose, fell sharply during 2012/2013 in the aftermath of a Medicines and Healthcare products Regulatory Agency (MHRA) safety alert issued in 2011.

**Conclusions:**

A relatively small percentage of these vulnerable patients are referred to mental health services, and reduced likelihood of referral in more deprived localities reflects a marked health inequality. National clinical guidelines have not yet been effective in reducing rates of tricyclic antidepressant prescribing for this high-risk group.

## Highlights

• The percentage of patients referred to mental health services is low.
• Patients in the most deprived localities have the least chance of being referred.
• One in ten are prescribed medication without a diagnosis or referral.
• Eleven percent have a diagnosis but are not medicated or referred.
• Despite guidance, a sizeable proportion were prescribed potentially toxic medication.

## 1 Introduction

Self-harm is one of the strongest risk factors for death by suicide (Cooper et al., 2007, Bergen et al., 2012) and general practitioners (GPs) play an important role in managing risk among patients who have recently harmed themselves. However, research evidence for the clinical management of self-harm specifically in primary care settings is lacking because most published studies have been conducted using hospital emergency department and admissions data. Nonetheless, the important role played by primary care in the assessment of people who self-harm was emphasised in 2004 by National Institute for Health and Care Excellence (NICE) clinical guideline 16: *Self-harm: the short-term physical and psychological management and secondary prevention of self-harm in primary and secondary care* (NICE, 2004). Despite this strong emphasis, just 14 of its 152 recommendations provided instruction to primary care teams, and this was the case for only 3 of the 57 recommendations made in 2011 by NICE clinical guideline 133: *Self-harm: longer-term management* (NICE, 2011). This lack of recommendations specific to primary care is linked to the absence of research evidence for this topic.

We examined a cohort extracted from the Clinical Practice Research Datalink (CPRD) (DoH, 2011; Herrett et al., 2015). This data source enabled us to examine primary care clinical management following an episode of self-harm using data from general practices located across the UK. The purpose was not to comprehensively ascertain all incident cases of self-harm in the population, including all cases treated in secondary care settings. Rather it was to investigate individuals whose recent self-harm episodes have been brought to the attention of their GPs. We initially profiled those patients who had an antecedent psychiatric diagnosis or a new one following their index self-harm episode. However, our primary outcomes were the percentage of cohort members receiving a referral to mental health services or prescribed psychotropic medication in the subsequent year. We paid particular attention to medication that can be fatally toxic in overdose, such as tricyclic antidepressants. Finally, we aimed to assess variability in clinical management by age and gender and by practice-level deprivation.

## 2 Methods

### 2.1 Description of the data source and study cohort

The December 2013 CPRD extract that we examined included 684 general practices and more than 13 million patients, with age and gender distributions comparable to those for the whole UK population (Herrett et al., 2015; García Rodríguez and Pérez Gutthann, 1998). Validation studies have reported consistently high CPRD quality data (Herrett et al., 2015, Khan et al., 2010). The Read code system (Chisholm, 1990), the standard for UK general practice, is routinely applied in the dataset. It provides a structured hierarchy of terms relating to demography and lifestyle, symptoms, diagnoses, therapies, referrals, and laboratory test results (HSCIC, 2015).

We delineated the study cohort using a broad definition that incorporated all forms of self-harm from the mildest non-suicidal episodes through to near-fatal attempted suicide, as described previously (Carr et al., 2016). Our definition excluded alcohol-related poisonings and suicidal ideation not involving actual self-harm acts. We initially identified potentially relevant Read codes using the search terms 'deliberate’, 'intent’ or 'self’ (to identify episodes of self-harm/harming, self-injury/injurious behaviour, self-inflicted harm/injury, harm/injury to self, self-poisoning, deliberate overdose, intentional overdose, etc.) and 'suicide attempt’, 'attempted suicide’ or 'parasuicide’ (to identify suicide attempts). The list of codes was then reviewed rigorously by two clinicians in the study team (NK and JC) and cross-referenced with a comparable list obtained from a recent CPRD-based validation study on suicide and self-harm (Thomas et al., 2013). Our final list can be downloaded from the 'ClinicalCodes.org’ repository (Springate et al., 2014).

An index self-harm episode was defined as the first occasion on which a Read code from our list was entered in a patient's clinical record. Limiting our extraction to patients deemed as being ‘up to standard’ for research purposes by the CPRD, our cohort consisted of individuals with a recorded index episode from 1st January 2001 to 31st December 2012. Patients were eligible for inclusion in a given year if they were aged 15–64 years and registered with a CPRD-contributing practice at the start of the year. The rationale for imposing these age restrictions was that the determinants and implications of self-harm in children and older adults are quite distinct from those of the rest of the population, and therefore warrant separate investigation and consideration. Among older persons who harm themselves, specific mechanisms such as bereavement, loneliness and social isolation (De Leo et al., 2001; Lebret et al., 2006) and physical illness, multi-morbidity and impairment (Lebret et al., 2006) play a predominant role; children aged below 15 years who harm themselves tend to have an unusually low suicidal intent and therefore a relatively low long-term risk of dying by suicide (Hawton and Harriss, 2008). To increase the likelihood that these were incident cases on entry into the study cohort, we stipulated that patients had to have been registered with a contributing CPRD practice on a continuous basis for at least a year prior to the index self-harm episode.

### 2.2 Classification and measurement

#### 2.2.1 Referrals and prescriptions

These were our two primary clinical management measures. We examined referrals to mental health services and psychotropic medication prescribing that was recorded subsequent to the index self-harm episode and during the 1 year follow-up period. We identified referrals to relevant mental health services using two CPRD fields. Firstly, a Family Health Services Authority (FHSA) variable indicated the department to which the patient was referred. General practitioners are required to enter this information upon referral, and for our purposes ‘Psychiatry’ was the only relevant department. Secondly, we also utilised the National Health Service (NHS) specialty field. This contains more granular information, but completion by general practice staff is not compulsory when coding referrals. The NHS specialty classification included eight mental health codes: mental illness; child and adolescent psychiatry; forensic psychiatry; psychotherapy; old age psychiatry; clinical psychology; adult psychiatry; and community psychiatric nurse. We combined information from both the FHSA and NHS fields to construct a binary specialist mental health services referral indicator. The dataset also contains complete records of all prescribed medication. We extracted all prescriptions in the following psychotropic medication classes: typical, atypical and depot antipsychotics; lithium and other mood stabilisers; selective serotonin reuptake inhibitor (SSRI), tricyclic and other antidepressants; benzodiazepines; opioid analgesics; other anxiolytics and hypnotics. Our list of Multilex product (FirstDataBank, 2014) codes for denoting psychotropic medications can be downloaded from ‘ClinicalCodes.org’ (Springate et al., 2014).

#### 2.2.2 Diagnoses

Psychiatric diagnoses were measured according to any prior history or a new diagnosis made after the index self-harm episode. They were classified as: schizophrenia-spectrum; bipolar disorder; depression; anxiety disorders; personality disorders; and eating disorders. Read code lists were compiled for each diagnostic category and were reviewed by two clinically qualified study team members (NK and JC). The final lists can be accessed at ‘ClinicalCodes.org’ (Springate et al., 2014); a rationale for these coding decisions is given in Supplemental file 1.

#### 2.2.3 Clinical consultation

The CPRD ‘consultation type’ field contains 59 categories, including numerous options that denote telephone consultations or administrative processes. A previous CPRD-based case-control study of death by suicide found that just eight of these categories were used in 96% of patient record entries (Appleby et al., 2014). As in that study, to provide a stringent measure of face-to-face contact with a GP or practice nurse, we applied categories 1 (‘clinic’) and 9 (‘surgery consultation’) only to derive our clinical consultation count variable.

#### 2.2.4 Deprivation

We applied an ecological measure at practice postcode level: the 2010 Indices of Multiple Deprivation (IMD) for England, Wales, Scotland and Northern Ireland.(Department for Communities and Local Government, 2012; The Scottish Government, 2012; The Welsh Government, 2010; Northern Ireland Statistics and Research Agency, 2010) The IMD reflects social and material deprivation among areas generally housing 1000–3000 residents, enabling rank ordering of area-level scores. We examined four separate quintile variables, which were generated from the continuous IMD scores, according to the distributions of the four UK nations.

### 2.3 Statistical analyses

All analyses were performed using Stata version 13 (StataCorp, 2013). We examined clinical events occurring within one year after the index self-harm episode. We stratified our analyses by gender, age in 10-year intervals and deprivation quintile. Due to the large cohort size and abundant statistical power, we found numerous instances of statistically significant heterogeneity by gender, age and deprivation albeit with only small absolute differences observed. Thus, we placed greater emphasis on the strength of association rather than p-values. We calculated 95% confidence intervals for binomial proportions using Wilson's method (Wilson, 1927) rather than applying a normal approximation, and we used Koopman's method to calculate the confidence interval for a ratio of two proportions (Koopman, 1984). The final cohort observation date was 31st December 2013. Therefore, patients with index episodes after 31st December 2012 were excluded because a full year of follow-up data was unavailable. We calculated the time elapsed from index self-harm episode to first recorded subsequent referral, only for those referrals occurring within a year of the index episode.

## 3 Results

### 3.1 Description of the study cohort

The full cohort consisted of 47,970 patients with an index self-harm episode during 2001–2012. The median follow-up time was 3.7 years (interquartile range: 1.7–6.8 years) and 41,500 (86.5%) patients had at least one full year of follow-up. Thus, 6470 patients (13.5%) did not complete the full follow-up year, with the percentage being higher in male patients than in females (15.5% versus 12.0%: Table 1). Almost a tenth (4,475; 9.3%, CI 9.1–9.6) of the total cohort with an index self-harm episode transferred to another practice during the follow-up year, and 1,052 (2.2%, 2.1–2.3) died; 4.0% (CI 3.7–4.2) of the male patients and 0.9% (CI 0.8–1.0) of the females died. Most of the 41,500 patients in the cohort with complete follow-up were female (24,317; 58.6%). They tended to be somewhat younger than their male counterparts, with a median age of 29 versus 31 years for males.

### 3.2 Psychiatric diagnoses, and subsequent referral and medication prescribing

**Fig. 1:**
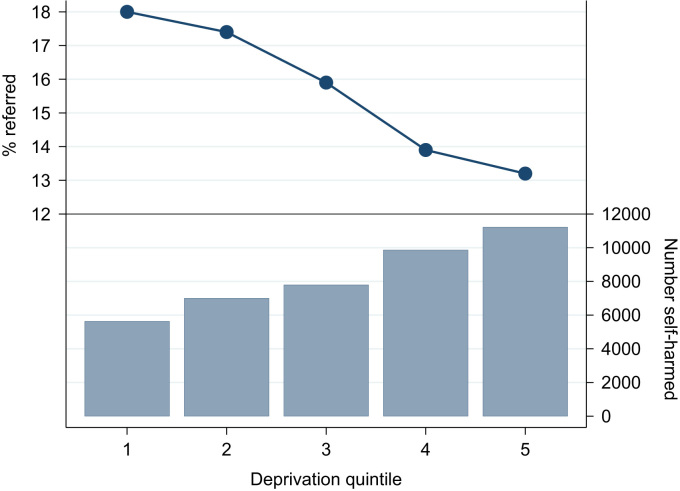
Percentage of patients referred to mental health services during the 1 year follow-up compared with number who self-harmed by deprivation quintile.

For the remainder of Section 3, we focus on the cohort members with a full year of follow up. Almost two thirds (26,389; 63.6%, CI 63.1–64.1) had an antecedent or new psychiatric diagnosis. Table 2 presents the percentages of patients who received psychotropic medication prescriptions or were referred to mental health services referrals in the year after their index episode: 6318 (15.2%, CI 14.9–15.6) were referred and 26,065 (62.8%, 62.3–63.3) were prescribed psychotropic medication. No strong gender differences in these percentages were apparent, but older patients of both genders were far more likely to receive a prescription after the index episode. Of the 6318 referrals recorded, the majority (3368; 53.3%) occurred within the first month of follow-up. Patients registered with practices in the most deprived localities were 27.1% (CI 21.5–32.2) less likely to be referred than those in the least deprived. Fig. 1 highlights the downward gradient in rates of referral to mental health services in relation to rising levels of deprivation, whilst the number of referrals increased incrementally from the least to the most deprived IMD quintile.

**Fig. 2:**
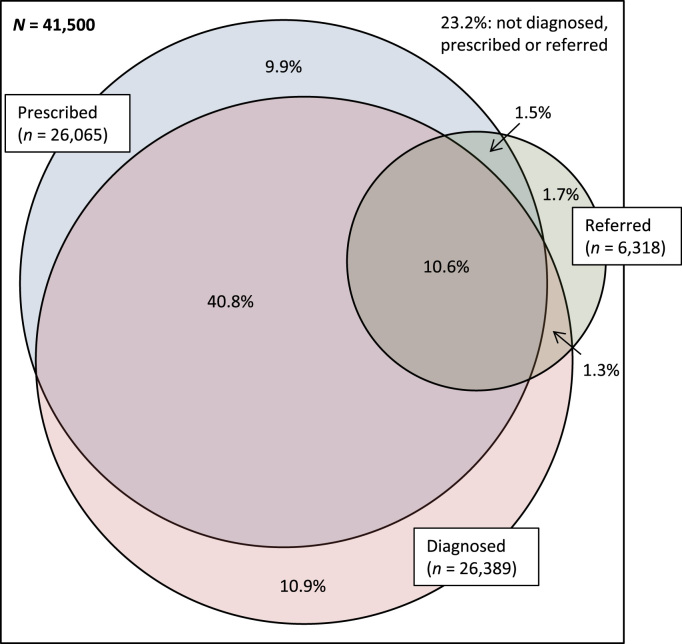
Venn diagram showing percentage values for patients with antecedent or new psychiatric diagnoses and with mental health service referrals or psychotropic drug prescriptions during the 1 year follow-up.

The Venn diagram shown in Fig. 2 depicts the percentages of cohort members who had antecedent or new psychiatric diagnoses, and who were referred or medicated during the follow-up year. 4105 (9.9%, CI 9.6–10.2) were prescribed psychotropic medication without a diagnosis or subsequent referral to mental health services, and 4506 (10.9%, CI 10.6–11.2) had a diagnosis but were not subsequently medicated or referred. Almost a quarter of cohort members (9648; 23.2%, CI 22.8–23.7) had no psychiatric diagnosis and were not subsequently referred or medicated.

In Supplemental file 2, we provide detailed information on diagnostic categories recorded at any time (historically or in the follow-up year combined), and new diagnoses made specifically during the 1 year follow-up. Consistent with prior expectation, the numerically dominant diagnostic groups in both genders were depression followed by anxiety disorders. Gender differences were modest for all diagnostic categories examined. In Table 3 we present the percentages of patients receiving prescriptions for individual psychotropic medication classes during the follow-up year. Predictably, SSRI antidepressants were the most frequently prescribed drug type, and other antidepressants, benzodiazepines, opioid analgesics and other anxiolytics/hypnotics were also commonly prescribed.

**Fig. 3:**
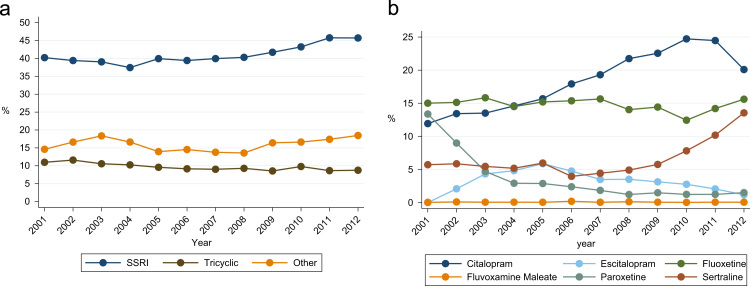
a. Temporal trends in the percentage of cohort members prescribed tricyclics, SSRIs and other antidepressants during the 1 year follow-up. 3b. Temporal trends in the percentage of cohort members prescribed particular types of SSRI antidepressants during the 1 year follow-up. *Footnote*: The x-axis values show the calendar year period when index self-harm episodes occurred. Each cohort member was followed up for 1 complete year. Thus, an individual born on 31st December 2012 was followed up to 31st December 2013, which was the final date of observation in our study.

Although they are known to be potentially fatally toxic in overdose, almost a tenth (3,985; 9.6%, CI 9.3–9.9) of cohort members were prescribed tricyclic antidepressant medication during the year after their index self-harm episode. Because of the clinical importance of this finding, we additionally examined temporal trends in SSRI versus tricyclic antidepressant prescribing. The trends plotted in Fig. 3a show increases in the percentages of cohort members prescribed SSRIs and other non-tricyclic antidepressant types during the follow-up year across the whole study period, but there was no compensatory fall over time in the percentage of patients prescribed a tricyclic. This percentage remained high throughout the 12 years of observation; it was 8.8% (CI 7.8–9.8) among patients whose index self-harm episode occurred during 2012 and who were followed up into 2013.

Fig. 3b plots temporal trends in the percentages of patients prescribed specific types of SSRIs. There was no discernible trend over the observation period in the percentages prescribed Fluoxetine or Fluvoxamine maleate. The percentage prescribed Paroxetine fell over time, especially in the earlier years of observation, and that for Sertraline increased sharply during the later years. Finally, the percentages prescribed Citalopram, and its S-enantiomer Escitalopram, fell over time; for Citalopram the percentage rose steadily across the study period until falling sharply in 2012/2013 with cohort members whose index self-harm episodes occurred during 2012.

We examined the characteristics of the patients in the study cohort who were prescribed tricyclic antidepressant medication. Of the 3985 patients prescribed a tricyclic antidepressant within a year of their index self-harm episode, 2466 (61.9%) were female and 64.8% were aged 35 years or older. Most patients (70.4%) had a diagnosis of depression prior to the date of their first tricyclic prescription during follow-up, and 10.4% had a diagnosis of depression recorded on the same day as this prescription was issued.

In Table A3 (in the online Supplemental material), we present the frequencies and percentage values for the following three measures:

1. *Ever prescribed an SSRI and/or other ADD at any time before first tricyclic prescription during follow-up.*
2. *Prescribed an SSRI and/or other ADD within a year prior to first tricyclic prescription during follow-up.*
3. *Prescribed an SSRI and/or other ADD between index self-harm episode and first tricyclic prescription during follow-up.*

The purpose of these analyses was to assess the degree to which tricyclic antidepressant medication was used as first-line treatment in the study cohort, as opposed to being a therapeutic approach that was taken only after SSRIs and/or other antidepressants had been prescribed. Among those prescribed tricyclics, 22.3% (95% CI 21.0–23.6%) had never been prescribed an SSRI and/or any other type of antidepressant, 39.2% (CI 37.7–40.7%) had not been prescribed these alternative antidepressant therapies within a year of first being prescribed tricyclics, and 64.5% (63.0–65.9%) had not been prescribed them between index self-harm episode and subsequent first tricyclic prescription. Among the subset of cohort members prescribed tricyclics, prior prescribing of an SSRI and/or another type of antidepressant medication was more common in female than in male patients.

### 3.3 Consultation patterns

The median consultation frequency during the year after the index episode was 7 visits (interquartile range: 3–12). Male patients consulted less frequently, with a median of 5 visits (interquartile range: 2–10), compared with a median among females of 8 visits (interquartile range: 4–13). Overall, 2961 (7.1%, CI 6.9–7.4) cohort members did not consult once during the follow-up year, with males being more likely to be non-attenders (1954; 11.4%, CI 10.9–11.9) than females (1007; 4.1%, CI 3.9–4.4).

## 4 Discussion

### 4.1 Key findings

For the first time, this study provides population-based evidence illustrating clinical management patterns among UK patients in primary care who have recently harmed themselves. Our findings indicate that general practices have major challenges to address if they are to provide optimal care for patients following self-harm. The most important findings were as follows:

1) Overall, the percentage of patients subsequently referred to mental health services was relatively low, and patients registered at practices in more deprived areas were less likely to be referred than those in less deprived areas.
2) Almost a tenth of cohort members were prescribed psychotropic medication without an antecedent or new psychiatric diagnosis or subsequent referral to mental health services, and nearly eleven percent had a diagnosis but were not subsequently medicated or referred.
3) A considerable proportion of patients, almost a tenth across the whole 12-year observation period and still nearly nine percent during 2012–2013, received potentially fatally toxic tricyclic antidepressant medication soon after harming themselves, and twenty-two percent of these patients had never been prescribed a different type of antidepressant drug previously.
4) The percentage of cohort members prescribed Citalopram fell sharply during 2012/2013.
5) Nearly a tenth of cohort members moved practices during the follow-up year and, among those patients with a complete year of follow-up, 7% did not consult with a GP or practice nurse in that period.

### 4.2 Comparison with existing evidence

With around 220,000 presentations of self-harm occurring annually (Hawton et al., 2007), much of the research evidence for self-harm in the UK and other developed countries has been generated through studies conducted using hospital emergency department and admissions data. Some evidence, does, however, exist for patterns of consultation in primary care following self-harm. One study found that most people consult with their GP soon after an episode, providing an opportunity to intervene and prevent further episodes (Gunnell et al., 2002). We found the overall rate of psychiatric referral to specialist mental health services in the year following self-harm to be around just 15% for all patients combined, which is lower than the 25% cited in relation to hospital-based presentations (Kapur et al., 2006). Given the complex health needs of this patient group, a higher referral rate by GPs might be expected. Because of the lack of relevant research we do not know what the optimal referral rate ought to be in this population and setting, but the apparently low rate is certainly a concern that merits further investigation. We have previously reported from the CPRD that patients registered at practices in more deprived localities have a higher incidence and annual presentation of self-harm (Carr et al., 2016). Thus, the decreasing referral rate with heightened levels of socioeconomic deprivation that we report here provides a clear illustration of Tudor Hart's ‘Inverse Care Law’ (Tudor Hart, 1971), whereby the provision of services is inversely associated with the level of needs in the population (Chew-Graham et al., 2002, Mercer and Watt, 2007).

Many previous studies have documented the severe and complex needs of people who have harmed themselves. This body of evidence indicates why these individuals require optimal clinical management in primary care. People who have self-harmed have a very high prevalence of mental illness, particularly depression and anxiety disorders in adults and attention deficit hyperactivity disorder (ADHD) and conduct disorder in adolescents (Hawton et al., 2013). They commonly have comorbid substance misuse and psychological distress (Moller et al., 2013), and they are also more likely to perpetrate violence and be victims of it (Vaughn et al., 2015). They are particularly prone to dying prematurely, specifically by suicide or accidental poisoning (Cooper et al., 2007, Bergen et al., 2012, Finkelstein et al., 2015a, Finkelstein et al., 2015b) and from alcohol-related causes (Bergen et al., 2014). In a cohort study conducted in three English cities, mean potential Years of Life Lost (YLL) for all-cause mortality were 31·4 years in males and 30·7 years in females (Bergen et al., 2012). As well as the apparent under-treatment of these patients, the prescribing of psychotropic medication without a psychiatric diagnosis is also potentially problematic, because it represents a departure from evidence-based clinical practice.

Careful prescribing is required for vulnerable primary care patients at elevated risk of self-poisoning (Hawton and Blackstock, 1976, Hawton and Blackstock, 1977) and in particular those individuals who have already harmed themselves. Monitoring patients in the early stages of treatment is therefore essential, as is balancing the risks of suicide and self-harm versus the efficacy of medications in treating the underlying mental illness (Gunnell et al., 2005). Many previous studies have reported on the potentially harmful effects of specific psychotropic medication classes. A comprehensive systematic review on the topic highlighted tricyclic antidepressants and certain types of selective serotonin reuptake inhibitor (SSRI) antidepressants as having heightened fatal toxicity risk in overdose (Flanagan, 2008). Other research has confirmed the toxicity of tricyclics (Hawton et al., 2010) and have identified potential risks in overdose associated with certain SSRIs (Gunnell et al., 2005), most notably Citalopram (Hawton et al., 2010, Barbui and Patten, 2014).

One investigation reported aggregated Primary Care Trust (PCT)-level antidepressant prescribing data in years 2004–2006 for three English cities, and patient-level data for emergency department self-poisoning cases at hospitals in these cities during 2000–2006 (Bergen et al., 2010). The authors described marked inter-PCT variability in antidepressant prescribing patterns and self-poisoning profiles with these drugs, and highlighted a specific concern regarding the continued prescribing of toxic tricyclics in this population. NICE clinical guideline 133 (CG133, recommendation 1.5.2; page 25) states: “When prescribing drugs for associated mental health conditions to people who self-harm, take into account the toxicity of the prescribed drugs in overdose. For example, when considering antidepressants, selective serotonin reuptake inhibitors (SSRIs) may be preferred because they are less toxic than other classes of antidepressants. In particular, do not use tricyclic antidepressants, such as dosulepin, because they are more toxic.” On the NICE CG133 website, recommendation 1.5.2 is flagged separately as being one of only three ‘*Do not do*’ recommendations from that guideline (https://www.nice.org.uk/guidance/cg133/resources/do-not-do). Although it was published in November 2011, we found no evidence of a marked decline in tricyclic prescribing for cohort members whose index self-harm episodes occurred during 2012 and were followed up into 2013. Of course this is a relatively short post-intervention time period, and it is feasible that prescription levels may have fallen subsequently. Nonetheless, we did observe a sharp fall during 2012/2013 in the percentage of patients who were prescribed Citalopram. This followed a Medicines and Healthcare products Regulatory Agency (MHRA) safety alert issued in December 2011 that focussed on cardiovascular risks (https://www.gov.uk/drug-safety-update/citalopram-and-escitalopram-qt-interval-prolongation).

Almost four fifths of patients who were prescribed a tricyclic antidepressant during follow-up after their index self-harm episode had been prescribed an SSRI or some other antidepressant drug agent previously. This indicates that tricyclics are generally not being selected as first-line treatment. Second-line use may seem a more clinically justifiable option as the self-harm event itself might suggest that the patient is not responding adequately well to earlier treatment with alternative antidepressant medications. That other antidepressant drugs have usually been tried previously adds useful insight into the challenges of clinical consultation in primary care, although twenty two percent of these patients appear to have been prescribed tricyclics in preference to alternative antidepressant agents.

### 4.3 Strengths and limitations

Approximately 6.9% of the UK population is registered at practices reporting data to the CPRD, which is broadly representative of the whole population nationally in terms of age, gender and ethnicity (Herrett et al., 2015). From this resource we extracted a large cohort to investigate a relatively rare phenomenon with abundant statistical power to examine subsequent primary care management profiles. Observational studies conducted in the CPRD rely on the standard of data inputted by GPs and other practice staff members. Data quality is, however, carefully monitored, and we restricted our analysis to those practices that were deemed to be up-to-standard for research purposes as defined by CPRD. The dataset conferred a number of specific advantages, including: (i) Diagnostic coding of a high standard (Khan et al., 2010); (ii) Mandatory recording of referrals to mental health services; and (iii) Recording of all medication prescribed in primary care. Our study did have some specific limitations. Firstly, we had no information on the uptake of referrals, and we could not determine whether a patient was already under the care of psychiatric services or, indeed, the date of commencement of such care. Secondly, psychological therapies and other non-medicinal treatments were not systematically recorded in the dataset. Finally, we assigned deprivation scores at practice level as proxies for patient-level measures.

## 5 Conclusions

Rates of referral to mental health services are relatively low among these patients, and a stark health inequality is evident in relation to a lower likelihood of being referred among patients registered at practices located in more deprived areas. A greater level of adherence to the 2011 NICE CG133 ‘*Do not do*’ recommendation against tricyclic antidepressant prescribing is also indicated. However, our findings also indicate that sizeable proportions of primary care patients move to another practice, or fail to consult with a GP or nurse, within a year of harming themselves. This represents a major challenge for general practices in monitoring these patients and in providing high quality care. Guidelines for GPs on managing patients with history of self-harm are currently limited, and further research is needed to determine how risks might be lowered through improved primary care clinical intervention.

## Figures and Tables

**Table 1:** Cohort members’ demographic information.

		**All**	**Male**	**Female**
Total cohort size (N)		47,970	20,325	27,645
Gender (%)		–	42.4	57.6
Median age (IQR)		30 (20,42)	32 (22,43)	28 (19,41)
			
**Patients with less than 1 year of follow-up:**				
Number of patients (*% of Total N*)		6470 (13.5)	3142 (15.5)	3328 (12.0)
Gender (row %)		–	48.6	51.4
Median age (IQR)		31 (21,42)	34 (24,45)	27 (19,39)
Lost to follow-up (*% of Total N*):				
	Died from any cause	2.1	3.8	0.9
	Transferred out of practice	9.3	9.7	9.0
	Practice no longer contributing to CPRD	2.1	1.9	2.2
			
**Patients with complete 1 year of follow-up:**				
Number of patients (*% of Total N*)		41,500 (86.5)	17,183 (84.5)	24,317 (88.0)
Gender (row %)		–	41.4	58.6
Median age (IQR)		30 (20,42)	31 (22,43)	29 (19,42)

**Table 2:** Mental health services referrals and psychotropic medication prescriptions during 1 year follow-up.

			**Total**	**Referred**		**Prescribed**	
			*N*	*n*	%	*n*	%
**Overall**			41,500	6,318	15.2	26,065	62.8
**Male**			17,183	2,467	14.4	10,405	60.6
	Age						
		15–24	5,925	813	13.7	2,397	40.5
		25–34	3,898	586	15.0	2,485	63.8
		35–44	3,702	557	15.1	2,698	72.9
		45–54	2,374	337	14.2	1,818	76.6
		55–64	1,284	174	13.6	1,007	78.4
	IMD quintile						
		1	2,182	383	17.6	1,318	60.4
		2	2,743	439	16.0	1,647	60.0
		3	3,185	488	15.3	1,969	61.8
		4	4,146	548	13.2	2,530	61.0
		5	4,927	609	12.4	2,941	59.7
**Female**			24,317	3,851	15.8	15,660	64.4
	Age						
		15–24	10,368	1,557	15.0	4,364	42.1
		25–34	4,348	780	17.9	3,293	75.7
		35–44	4,957	841	17.0	4,069	82.1
		45–54	3,255	497	15.3	2,754	84.6
		55–64	1,389	176	12.7	1,180	85.0
	IMD quintile						
		1	3,444	632	18.4	2,207	64.1
		2	4,255	776	18.2	2,771	65.1
		3	4,610	752	16.3	2,939	63.8
		4	5,718	824	14.4	3,698	64.7
		5	6,290	867	13.8	4,045	64.3

Patients that did not complete one year of follow-up were excluded

IMD quintile 1 denotes least deprived and quintile 5 most deprived

**Table 3:** Psychotropic prescribing by type during 1 year follow-up.

	**All** (*N*=41,500)		**Male** (*N*=17,183)		**Female** (*N*=24,317)	
**Psychotropic medication**	***n***	**%**	***n***	**%**	***n***	**%**
Typical antipsychotics	2,292	5.5	773	4.5	1,519	6.3
Atypical antipsychotics	3,319	8.0	1,556	9.1	1,763	7.3
Depot antipsychotics	72	0.2	31	0.2	41	0.2
Lithium and other mood stabilisers	1,901	4.6	787	4.6	1,114	4.6
SSRI antidepressants	17,030	41.0	6,280	36.6	10,750	44.2
Tricyclic antidepressants	3,985	9.6	1,519	8.8	2,466	10.1
Other antidepressants	6,580	15.9	2,586	15.1	3,994	16.4
Benzodiazepines	7,637	18.4	3,139	18.3	4,498	18.5
Opioid analgesics	5,605	13.5	2,252	13.1	3,353	13.8
Other anxiolytics and hypnotics	5,592	13.5	2,201	12.8	3,391	13.9
**Any psychotropic medication**	26,065	62.8	10,405	60.6	15,660	64.4

All analyses restricted to patients with at least one year of follow-up.

## References

[bib1] Appleby L., Kapur N., Shaw J., Windfuhr K., While D., Webb R., Ashcroft D., Kontopantelis E. (2014). Suicide in Primary Care in England: 2002-2011. National Confidential Inquiry Into Suicide and Homicide by (People) (with Mental Illness (NCISH).

[bib2] Barbui C., Patten S.B. (2014). Antidepressant dose and the risk of deliberate self-harm. Epidemiol. Psychiatr. Sci..

[bib3] Bergen H., Murphy E., Cooper J., Kapur N., Stalker C., Waters K., Hawton K. (2010). A comparative study of non-fatal self-poisoning with antidepressants relative to prescribing in three Centres in England. J. Affect. Disord..

[bib5] Bergen H., Hawton K., Waters K., Ness J., Cooper J., Steeg S., Kapur N. (2012). Premature death after self-harm: a multicentre cohort study. Lancet.

[bib6] Bergen H., Hawton K., Webb R., Cooper J., Steeg S., Haigh M., Ness J., Waters K., Kapur N. (2014). Alcohol-related mortality following self-harm: a multicentre cohort study. J. R. Soc. Med. Open.

[bib4] Carr M.J, Ashcroft D.M., Kontopantelis E., Awenat Y., Cooper J., Chew-Graham C., Kapur N., Webb R.T. (2016). The epidemiology of self-harm in the UK primary care patient population, 2001-2013. BMC Psychiatry.

[bib7] Chew-Graham C.A., Mullin S., May C.R., Hedley S., Cole H. (2002). Managing depression in primary care: another example of the inverse care law?. Fam. Pract..

[bib8] Chisholm J. (1990). The Read clinical classification. BMJ.

[bib9] Cooper J., Kapur N., Webb R., Lawlor M., Guthrie E., Mackway-Jones K., Appleby L. (2007). Suicide after deliberate self-harm: a 4-year cohort study. Am. J. Psychiatry.

[bib10] De Leo D., Padoani W., Scocco P., Lie D., Bille-Brahe U., Arensman E., Hjelmeland H., Crepet P., Haring C., Hawton K., Lonnqvist J., Michel K., Pommereau X., Querejeta I., Phillipe J., Salander-Renberg E., Schmidtke A., Fricke S., Weinacker B., Tamesvary B., Wasserman D., Faria S. (2001). Attempted and completed suicide in older subjects: results from the WHO/EURO multicentre study of suicidal behaviour. J. Geriatr. Psychiatry.

[bib11] Department for Communities and Local Government (2010). English Indices of Deprivation. http://https://www.gov.uk/government/collections/english-indices-of-deprivation.

[bib12] Department of Health (2011). Launch of the Clinical Practice Research Data Link.

[bib13] Finkelstein Y., Macdonald E.M., Hollands S., Hutson J.R., Sivilotti M.L.A., Mamdani M.M., Koren G., Juurlink D.N. (2015). Long-term outcomes following self-poisoning in adolescents: a population-based cohort study. Lancet Psychiatry.

[bib14] Finkelstein Y., Macdonald E.M., Hollands S., Sivilotti M.L.A., Hutson J.R., Mamdani M.M., Koren G., Juurlink D.N. (2015). Risk of suicide following deliberate self-poisoning. JAMA Psychiatry.

[bib15] FirstDataBank (2014). MULTILEX. http://www.fdbhealth.co.uk/solutions/multilex/.

[bib16] Flanagan R.J. (2008). Fatal toxicity of drugs used in psychiatry. Hum. Psychopharmacol..

[bib17] García Rodríguez L.A., Pérez Gutthann S. (1998). Use of the UK General Practice Research Database for pharmacoepidemiology. Br. J. Clin. Pharmacol..

[bib18] Gunnell D., Saperia J., Ashby D. (2005). Selective serotonin reuptake inhibitors (SSRIs) and suicide in adults: meta-analysis of drug company data from placebo controlled, randomised controlled trials submitted to the MHRA's safety review. BMJ.

[bib19] Gunnell D., Bennewith O., Peters T.J., Stocks N., Sharp D.J. (2002). Do patients who self-harm consult their general practitioner soon after Hospital discharge? A cohort study. Soc. Psychiatry Psychiatr. Epidemiol..

[bib20] Hawton K., Blackstock E. (1976). General practice aspects of self-poisoning and self-injury. Psychol. Med..

[bib21] Hawton K., Blackstock E. (1977). Deliberate self-poisoning: implications for psychotropic drug prescribing in general practice. J. R. Coll. Gen. Pract..

[bib22] Hawton K., Harriss L. (2008). Deliberate self-harm by under 15-year-olds: characteristics, trends and outcome. J. Child. Psychol. Psychiatry.

[bib23] Hawton K., Saunders K., Topiwala A., Camilla H. (2013). Psychiatric disorders in patients presenting to hospital following self-harm: a systematic review. J. Affect. Disord..

[bib24] Hawton K., Bergen H., Simkin S., Cooper J., Waters K., Gunnell D., Kapur N. (2010). Toxicity of antidepressants: rates of suicide relative to prescribing and non-fatal overdose. Br. J. Psychiatry.

[bib25] Hawton K., Bergen H., Casey D., Simkin S., Palmer B., Cooper J., Kapur N., Horrocks J., House A., Lilley R., Noble R., Owens D. (2007). Self-harm in England: a tale of three cities. Multicentre study of self-harm. Soc. Psychiatry Psychiatr. Epidemiol..

[bib26] Health and Social Care Information Centre (HSCIC) (2015). NHS UK Read Codes Clinical Terms Version 3. http://https://isd.hscic.gov.uk/trud3/user/guest/group/0/pack/9.

[bib27] Herrett E., Gallagher A.M., Bhaskaran K., Forbes H., Mathur R., van Staa T., Smeeth L. (2015). Data resource profile: Clinical Practice Research Datalink (CPRD). Int. J. Epidemiol..

[bib28] Kapur N., Cooper J., King-Hele S., Webb R., Lawlor M., Rodway C., Appleby L. (2006). The repetition of suicidal behavior: a multicenter cohort study. J. Clin. Psychiatry.

[bib29] Khan N.F., Harrison S.E., Rose P.W. (2010). Validity of diagnostic coding within the General Practice Research Database: a systematic review. Br. J. Gen. Pract..

[bib30] Koopman P.A. (1984). Confidence intervals for the ratio of two binomial proportions. Biometrics.

[bib31] Lebret S., Perret-Vaille E., Mulliez A., Gerbaud L., Jalenques I. (2006). Elderly suicide attempters: characteristics and outcome. Int. J. Geriatr. Psychiatry.

[bib32] Mercer S.W., Watt G. (2007). The inverse care law: clinical primary care encounters in deprived and affluent areas of Scotland. Ann. Fam. Med..

[bib33] Moller C.I., Tait R.J., Byrne D.G. (2013). Self-harm, substance misuse and psychological distress in the Australian general population. Addiction.

[bib34] NHS National Institute for Health and Care Excellence (NICE (2004). Self-harm: the short-term physical and psychological management and secondary prevention of self-harm in primary and secondary care. NICE Clin. Guidel..

[bib35] NHS National Institute for Health and Care Excellence (NICE) (2011). Self-harm: longer-term management. NICE Clin. Guidel..

[bib36] Northern Ireland Statistics and Research Agency (2010). Northern Ireland Multiple Deprivation Measure. http://www.nisra.gov.uk/deprivation/nimdm_2010.htm.

[bib37] Springate D.A., Kontopantelis E., Ashcroft D.M., Olier I., Parisi R., Chamapiwa E., Reeves D. (2014). ClinicalCodes: an online clinical codes repository to improve the validity and reproducibility of research using electronic medical records. PLoS One.

[bib38] StataCorp, 2013. Stata statistical software: release 13.

[bib39] The Scottish Government, 2012. Scottish Index of Multiple Deprivation (SIMD) 〈http://www.gov.scot/Topics/Statistics/SIMD〉.

[bib40] Thomas K.H., Davies N., Metcalfe C., Windmeijer F., Martin R.M., Gunnell D. (2013). Validation of suicide and self-harm records in the clinical practice research datalink. Br J. Clin. Pharmacol..

[bib41] Tudor Hart J. (1971). The inverse care law. Lancet.

[bib42] Vaughn M.G., Salas-Wright C.P., DeLisi M., Larson M. (2015). Deliberate self-harm and the nexus of violence, victimization, and mental health problems in the United States. Psychiatr. Res..

[bib43] Welsh Government (2010). The Welsh Index of Multiple Deprivation (WIMD). http://wales.gov.uk/statistics-and-research/welsh-index-multiple-deprivation.

[bib44] Wilson E.B. (1927). Probable inference, the law of succession, and statistical inference. J. Am. Stat. Assoc..

