# Supplementary material for: Clinical management following self-harm in a UK-wide primary care cohort
Source: J Affect Disord. 2016 Jun;197:182–8. doi: 10.1016/j.jad.2016.03.013 (PMC4870375; doi:10.1016/j.jad.2016.03.013)
Supplement: Supplementary material [file mmc1.docx]

**Supplemental file 1: Explanation of psychiatric diagnostic classifications**

Our schizophrenia-spectrum definition included diagnoses of delusional disorders, brief psychotic disorders, schizophreniform disorders, schizoaffective disorders, and schizophrenia. The spectrum definition also included patients with psychotic symptoms that did not adequately fit the definition for any of the specified diagnostic subgroups. We identified patients with bipolar disorder via records that referred directly to bipolar or affective disorder and records describing recurrent mania, manic-depression, or depression with psychosis. We applied broad definitions for the two most common conditions: depression and anxiety. Our definition of depression included the full range of diagnoses from either single or recurrent episodes of mild to severe depression through to more persistent chronic conditions. Anxiety incorporated panic disorders, phobias, post-traumatic stress disorders, social anxiety disorders, and generalised anxiety disorder.

Defining personality disorders can be complex due to the substantial overlap with other diagnostic categories. This is particularly true when considering variants that include some form of paranoia or psychosis. For instance, some definitions place schizoid and schizotypal personality disorders in the schizophrenia spectrum whereas our definition included these conditions as personality disorders when references to delusions or hallucinations were not present in the patient’s records. Our definition also incorporated erratic variants (including antisocial, borderline, histrionic, and narcissistic personality disorders) and anxiety or stress-related variants (including avoidant, dependent, and obsessive-compulsive personality disorders). The classification of eating disorders is also prone to subjectivity. Again, we applied a broad definition that covered the more common conditions (including anorexia nervosa, bulimia, and binge eating) but also included some of the rarer psychogenic variants.
