# Supplementary material for: Clinical management following self-harm in a UK-wide primary care cohort
Source: J Affect Disord. 2016 Jun;197:182–8. doi: 10.1016/j.jad.2016.03.013 (PMC4870375; doi:10.1016/j.jad.2016.03.013)
Supplement: Supplementary material [file mmc2.docx]

**Supplemental file 2: Psychiatric diagnostic categories**

| **Table A1: Psychiatric diagnostic categories for diagnoses made**  **at any time prior to or within 1 year of index self-harm episode** | | | | | | |
| --- | --- | --- | --- | --- | --- | --- |
|  | | | | | | |
| **Diagnostic category (%)** | **All**  (*N =* 41,500) | | **Male**  (*N =* 17,183) | | **Female**  (*N =* 24,317) | |
|  | ***n*** | **%** | ***n*** | **%** | ***n*** | **%** |
|  | | | | | | |
| Schizophrenia spectrum | 1,873 | 4.5 | 1,055 | 6.1 | 818 | 3.36 |
| Bipolar disorder | 981 | 2.4 | 341 | 2.0 | 640 | 2.6 |
| Depression | 22,033 | 53.1 | 8,160 | 47.5 | 13,873 | 57.1 |
| Personality disorders | 1,143 | 2.8 | 532 | 3.1 | 611 | 2.5 |
| Eating disorders | 1,493 | 3.6 | 207 | 1.2 | 1,286 | 5.3 |
| Anxiety disorders | 14,930 | 36.0 | 5,461 | 31.8 | 9,469 | 38.9 |
| **Any mental illness** | 26,389 | 63.6 | 10,128 | 58.9 | 16,261 | 66.9 |
|  | | | | | | |
| - Patients that did not complete one year of follow-up were excluded | | | | | | |

| **Table A2: Psychiatric diagnostic categories for new diagnoses made**  **during the 1 year follow-up** | | | | | | |
| --- | --- | --- | --- | --- | --- | --- |
|  | | | | | | |
| **Diagnostic category (%)** | **All**  (*N =* 41,500) | | **Male**  (*N =* 17,183) | | **Female**  (*N =* 24,317) | |
|  | ***n*** | **%** | ***n*** | **%** | ***n*** | **%** |
|  | | | | | | |
| Schizophrenia spectrum | 713 | 1.7 | 409 | 2.4 | 304 | 1.3 |
| Bipolar disorder | 363 | 0.9 | 104 | 0.6 | 259 | 1.1 |
| Depression | 9,875 | 23.8 | 3,715 | 21.6 | 6,160 | 25.3 |
| Personality disorders | 483 | 1.2 | 201 | 1.2 | 282 | 1.2 |
| Eating disorders | 360 | 0.9 | 52 | 0.3 | 308 | 1.3 |
| Anxiety disorders | 4,850 | 11.7 | 1,790 | 10.4 | 3,060 | 12.6 |
| **Any mental illness** | 13,154 | 31.7 | 5,039 | 29.3 | 8,115 | 33.4 |
|  | | | | | | |
| - Patients that did not complete one year of follow-up were excluded | | | | | | |
