# Supplementary material for: Clinical management following self-harm in a UK-wide primary care cohort
Source: J Affect Disord. 2016 Jun;197:182–8. doi: 10.1016/j.jad.2016.03.013 (PMC4870375; doi:10.1016/j.jad.2016.03.013)
Supplement: Supplementary material [file mmc3.docx]

**Table A3: Prior SSRI and other antidepressant drug (ADD) prescribing among patients in the study cohort who received a tricyclic antidepressant drug prescription within a year following their index self-harm episode**

|  |  |  | |  | |  | |
| --- | --- | --- | --- | --- | --- | --- | --- |
| **Prior SSRI and other ADD prescribing** |  | **a) SSRI:** | | **b) Other ADD:** | | **c) SSRI and/or other ADD:** | |
|  | ***N*** | ***n*** | **%** | ***n*** | **%** | ***n*** | **%** |
|  |  |  |  |  |  |  |  |
|  |  |  |  |  |  |  |  |
| *1. Ever prescribed an SSRI and/or other ADD at any time before first tricyclic prescription during follow-up* |  |  |  |  |  |  |  |
|  |  |  |  |  |  |  |  |
| All patients | 3985 | 2938 | 73.7 | 1382 | 34.7 | 3096 | 77.7 |
| Males | 1519 | 1031 | 67.9 | 454 | 29.9 | 1098 | 72.3 |
| Females | 2466 | 1907 | 77.3 | 928 | 37.6 | 1998 | 81.0 |
|  |  |  |  |  |  |  |  |
|  |  |  |  |  |  |  |  |
| *2. Prescribed an SSRI and/or other ADD within a year prior to first tricyclic prescription during follow-up* |  |  |  |  |  |  |  |
|  |  |  |  |  |  |  |  |
| All patients | 3985 | 2019 | 50.7 | 901 | 22.6 | 2423 | 60.8 |
| Males | 1519 | 698 | 46.0 | 306 | 20.1 | 835 | 55.0 |
| Females | 2466 | 1321 | 53.6 | 595 | 24.1 | 1588 | 64.4 |
|  |  |  |  |  |  |  |  |
|  |  |  |  |  |  |  |  |
| *3. Prescribed an SSRI and/or other ADD between index self-harm episode and first tricyclic prescription during follow-up* |  |  |  |  |  |  |  |
|  |  |  |  |  |  |  |  |
| All patients | 3985 | 1081 | 27.1 | 512 | 12.8 | 1416 | 35.5 |
| Males | 1519 | 406 | 26.7 | 169 | 11.1 | 504 | 33.2 |
| Females | 2466 | 675 | 27.4 | 343 | 13.9 | 912 | 37.0 |
|  |  |  |  |  |  |  |  |
